# Climate mitigation outcomes from China-led emission reductions toward global carbon neutrality

**DOI:** 10.1093/nsr/nwaf545

**Published:** 2025-12-09

**Authors:** Yadong Lei, Zhili Wang, Junting Zhong, Xiaochao Yu, Lifeng Guo, Chenguang Tian, Lei Li, Yixiong Lu, Da Zhang, Lin Liu, Deying Wang, Huizheng Che, Xiaoye Zhang

**Affiliations:** State Key Laboratory of Severe Weather Meteorological Science and Technology & Key Laboratory of Atmospheric Chemistry of CMA, Chinese Academy of Meteorological Sciences, Beijing 100081, China; State Key Laboratory of Severe Weather Meteorological Science and Technology & Key Laboratory of Atmospheric Chemistry of CMA, Chinese Academy of Meteorological Sciences, Beijing 100081, China; State Key Laboratory of Severe Weather Meteorological Science and Technology & Key Laboratory of Atmospheric Chemistry of CMA, Chinese Academy of Meteorological Sciences, Beijing 100081, China; State Key Laboratory of Severe Weather Meteorological Science and Technology & Key Laboratory of Atmospheric Chemistry of CMA, Chinese Academy of Meteorological Sciences, Beijing 100081, China; State Key Laboratory of Severe Weather Meteorological Science and Technology & Key Laboratory of Atmospheric Chemistry of CMA, Chinese Academy of Meteorological Sciences, Beijing 100081, China; School of Environmental Science and Engineering, Nanjing University of Information Science & Technology, Nanjing 210044, China; State Key Laboratory of Severe Weather Meteorological Science and Technology & Key Laboratory of Atmospheric Chemistry of CMA, Chinese Academy of Meteorological Sciences, Beijing 100081, China; CMA Earth System Modeling and Prediction Centre, China Meteorological Administration, Beijing 100081, China; Tsinghua-CTG Joint Center for Climate Governance and Low-carbon Transformation, Tsinghua University, Beijing 100086, China; State Key Laboratory of Severe Weather Meteorological Science and Technology & Key Laboratory of Atmospheric Chemistry of CMA, Chinese Academy of Meteorological Sciences, Beijing 100081, China; State Key Laboratory of Severe Weather Meteorological Science and Technology & Key Laboratory of Atmospheric Chemistry of CMA, Chinese Academy of Meteorological Sciences, Beijing 100081, China; State Key Laboratory of Severe Weather Meteorological Science and Technology & Key Laboratory of Atmospheric Chemistry of CMA, Chinese Academy of Meteorological Sciences, Beijing 100081, China; State Key Laboratory of Severe Weather Meteorological Science and Technology & Key Laboratory of Atmospheric Chemistry of CMA, Chinese Academy of Meteorological Sciences, Beijing 100081, China

**Keywords:** warming level, climate mitigation outcomes, NDCs, emissions scenario

## Abstract

While many nations committed to the Paris Agreement have completed the second-round updates to their nationally determined contributions (NDCs), especially China’s dual carbon commitment, the specific climate outcomes of these latest NDCs remain uncertain. Here, we quantify the potential climate mitigation outcomes from these latest NDCs through ensemble simulations of an Earth System Model under a newly developed global emission scenario aligned with China’s carbon neutrality pathway. We project a global temperature rise of 2.05°C during 2081–2100 through the implementation of the latest NDCs, demonstrating a likely achievable 2.0°C target without early extensive carbon removal technologies. Moreover, our results demonstrate that the latest NDCs will yield significant long-term climate benefits while incurring adverse near-term impacts, revealing temporally asymmetric climate outcomes when compared to fixing anthropogenic emissions at 2023 levels. We believe this work is valuable for understanding more plausible future climate change, with particular relevance to the ongoing seventh assessment report of the Intergovernmental Panel on Climate Change (IPCC).

## Teaser

This work quantifies the potential climate mitigation outcomes from the latest Nationally Determined Contributions under a newly-developed global emission scenario aligned with China’s carbon neutrality pathway, demonstrating a likely achievable 2.0Â°C target without early extensive carbon removal technologies.

## INTRODUCTION

Greenhouse gas (GHG) emissions from human activities have contributed to a continued warming climate with an increase in global mean surface temperature (GMST) by 1.09°C from 1850–1900 to 2011–20 [1]. Climate extremes such as heatwaves, floods, wildfires and droughts have become more frequent and co-occurrent in a warmer world [2–4], posing a serious threat to ecosystems and human society [5]. If GHG emissions remain at current levels or continue to increase, global warming is likely to reach 3°C–5°C by the end of the 21st century [6], which could trigger multiple climate tipping points such as Amazon dieback, boreal forest shifts and large-scale permafrost collapse [7].

To address climate change challenges, more than 120 nations have committed to achieving carbon neutrality to strive for the 2°C warming target of the 2015 Paris Agreement [8,9], and most of them have formulated detailed plans through laws or policy documents in the second-round nationally determined contributions (NDCs) in 2021. For example, as a major emitter accounting for 31% of global carbon emissions in 2023 [10], China proposed dual carbon goals, with a carbon peak before 2030 and carbon neutrality before 2060, which serve as the main driver for achieving global carbon neutrality; The European Union formulated Climate Law to cut GHG emissions by >55% compared to 1990 by 2030 and achieve climate neutrality by 2050 [11]; the USA pledged to achieve net-zero GHG emissions by 2050 [12]. An urgent question is, what are the potential climate outcomes of these updated NDCs around the world?

The climate change projections depend on future emission scenarios [13]. However, the widely used Shared Socioeconomic Pathways (SSP) scenarios supporting the sixth assessment report (AR6) of the Intergovernmental Panel on Climate Change (IPCC) used 2015 as the base year [14]. In the past decade, global GHG and pollutant emissions have experienced unprecedented changes. For example, China’s clean air actions decreased national SO_2_ by 69% while achieving co-benefits of a net accumulative CO_2_ decrease of 2.43 Gt during 2013–20 [15]. The COVID-19 pandemic resulted in a remarkable global reduction of 6% in 2020 emissions [16]. Although a modifying scenario based on SSP2-4.5 was developed for CovidMIP by accounting for the COVID-19 pandemic and some updated NDCs [17], it still has some limitations: (i) it fails to consider China’s dual carbon goals, while China accounts for 31% of global carbon emissions in 2023 [10]; and (ii) this scenario ends in the mid-21st century, but the Paris Agreement focuses on the warming level by the end of the 21st century. Therefore, these limitations in historical emissions and future scenarios hinder the accurate assessments of climate benefits from these latest updated NDCs around the world.

Here, based on updated historical emissions and a newly developed China-aligned global emissions scenario (named SSP2-com: accounting for up-to-date anthropogenic emissions and latest updated NDCs, including China’s dual carbon pledge), we present a more plausible global climate change projection from three ensemble transient simulations of the Community Earth System Model Version 2 (CESM2). Moreover, in combination with a baseline scenario (named Fix2023: all anthropogenic emissions during 2023–2100 are fixed at the 2023 level), close to the medium scenario in the ScenarioMIP-CMIP7 [18], we further quantify the potential outcomes of climate change mitigation from the latest updated NDCs around the world by the end of the 21st century. We believe this work will provide a useful fundamental dataset for the assessments relevant to climate change and valuable support for the ongoing IPCC AR7.

## RESULTS

### CO_2_ and aerosol optical depth (AOD)

**Figure 1.**
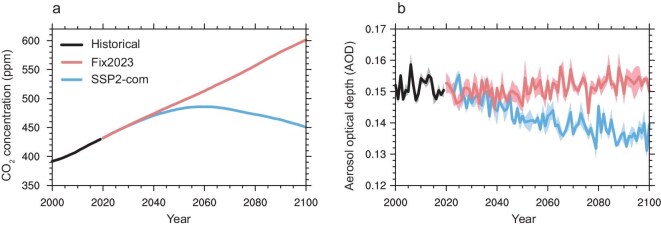
Simulated (a) global mean near-surface CO_2_ concentration and (b) land mean AOD during 2020–2100 under Fix2023 and SSP2-com scenarios. The shading represents one standard deviation of three ensemble simulations.

The climate change projection in the Coupled Model Intercomparison Project Phase 6 (CMIP6) is typically driven by zonally distributed CO_2_ concentrations [19]. Our simulations use monthly gridded CO_2_ emissions to drive CESM2, which further account for the spatial effects of CO_2_ on climate change. The simulated near-surface CO_2_ concentrations and AOD in the visible spectrum (550 nm) show large discrepancies under SSP2-com and Fix2023 scenarios (Fig. 1). Under the Fix2023 scenario, the simulated global mean CO_2_ concentration continues to rise to 601 ppm in 2100, increasing by 169 ppm from 2020 to 2100 (Fig. 1a). In contrast, the simulated global mean CO_2_ concentration peaks at 486 ppm in 2058, and then declines to 450 ppm in 2100 in the SSP2-com scenario. This demonstrates that global CO_2_ concentration will peak as expected in the mid-21st century if all the latest updated NDCs around the world are fully implemented. Following fixed anthropogenic emissions in 2023, the simulated global mean AOD over land shows limited changes from 2020 to 2100 (Fig. 1b). In contrast, the simulated global mean AOD over land exhibits a large decline from 2020 to 2070 due to sharp aerosol emission reductions, and remains relatively stable from 2070 to 2100 under the SSP2-com scenario. Spatially, the largest decline in AOD occurs in China, exceeding 50% under the SSP2-com scenario due to deep anthropogenic emission reductions driven by China’s dual carbon goals (Fig. S1).

### Temperature and precipitation

**Figure 2.**
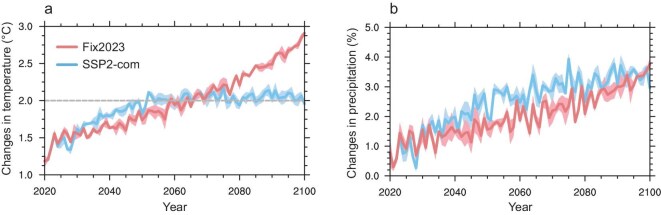
(a) Simulated global mean surface air temperature anomalies relative to 1850–1900 during 2020–2100 under Fix2023 and SSP2-com scenarios. (b) Simulated global mean precipitation anomalies during 2020–2100 under Fix2023 and SSP2-com scenarios, relative to the historical period (2000–19). The shading represents one standard deviation of three ensemble simulations.

The future changes in GHG and aerosol emissions significantly affect global temperature and precipitation (Fig. 2). The warming level by the end of the 21st century is the most concerning scientific issue for the IPCC. Using three very simplified climate emulators, Zhong *et al.* reported a temperature rise of 2.04°C during 2091–2100 under the SSP2-com scenario [20]. Here, we assess the temperature rise by the end of the 21st century under the SSP2-com scenario based on three ensemble transient simulations from the fully coupled CESM2 (for details see the Methods section). The simulated ensemble-mean GMST rise rapidly reaches up to 2°C in the mid-21st century, and then remains relatively stable with a temperature rise of 2.05°C during 2081–2100 under the SSP2-com scenario (Fig. 2a). This estimate of warming level in CESM2 is consistent with previous estimate of 2.04°C using three very simplified climate emulators [20]. It is noted that the CESM2 model has an equilibrium climate sensitivity (ECS, defined as the global mean surface warming in response to a doubling of atmospheric CO_2_) of 5.3 K [21], higher than the best estimate of ∼3.0 K in IPCC AR6. Given the low ECS in most other ESMs, it is highly likely that the well below 2°C GMST target of the Paris Agreement can be achieved if all the latest updated NDCs around the world are fully implemented (represented by the SSP2-com scenario).

However, the GMST will increase by 2.91°C in 2100 with a temperature rise of 2.57°C during 2081–2100 if anthropogenic emissions remain fixed at the level of 2023 in the Fix2023 scenario. It is noted that although the mitigation efforts from the latest updated NDCs bring strong global cooling effects (e.g. 2.05°C vs. 2.57°C for warming level) in the late 21st century, this climate benefit is not found in the early and mid-21st century. On the contrary, the simulated GMST from 2023 to 2060 under the SSP2-com scenario is higher than that under the Fix2023 scenario. This lagged temperature benefit from nationally determined mitigation efforts can be attributed to the opposite effects of GHG and aerosol emission reductions on global warming. CO_2_ mitigation can decrease global temperature, but the co-sourced aerosol emission reductions in the early and mid-21st century cause a strong heating effect on Earth due to enhanced solar radiation energy. The warming in the mid to high latitudes of the Northern Hemisphere driven by aerosol emission reductions are also reported in the COVID-19 pandemic period [22].

**Figure 3.**
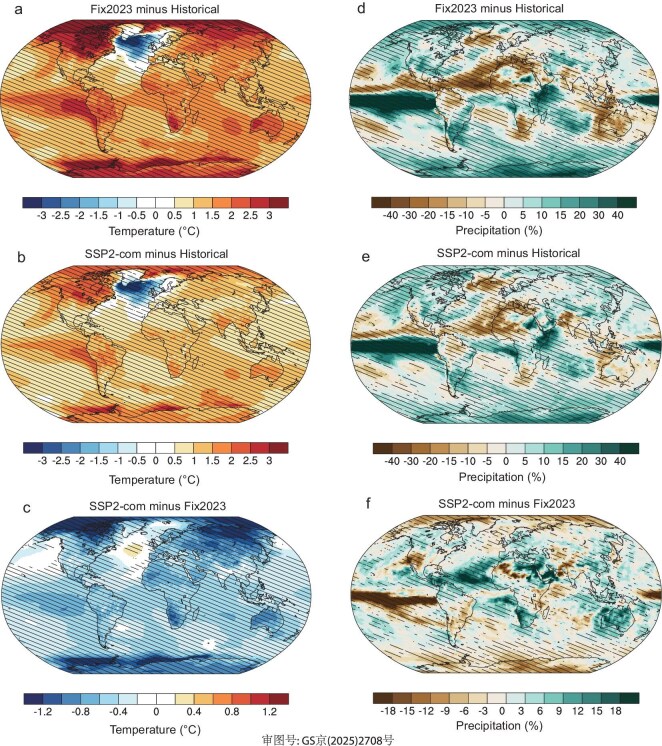
(a and b) Simulated annual mean changes of surface air temperature by the end of the 21st century (2081–2100) under Fix2023 (a) and SSP2-com (b) scenarios relative to the historical period. (d and e) Simulated annual mean changes of precipitation by the end of the 21st century under Fix2023 (d) and SSP2-com (e) scenarios, relative to the historical period. (c and f) The differences of simulated annual mean air temperature (c) and precipitation (f) by the end of the 21st century between SSP2-com and Fix2023 scenarios (SSP2-com minus Fix2023). The hatched regions represent that all three ensemble simulations agree on the direction of changes.

Spatially, the warming in both Fix2023 and SSP2-com scenarios by the end of the 21st century over land regions is larger than that over ocean regions (Fig. 3a and b). The largest warming in both Fix2023 and SSP2-com scenarios by the end of the 21st century is found in the mid-to-high latitudes, especially in the Arctic. This phenomenon, named polar amplification, is also seen in instrumental observations [23], which is attributed to multiple factors (e.g. surface albedo feedback and reduced air pollution). Moreover, the North Atlantic Ocean, named the North Atlantic warming hole [24], experiences further cooling by >1°C by the end of the 21st century under both Fix2023 and SSP2-com scenarios. Although the warming in both Fix2023 and SSP2-com scenarios shows similarities in their spatial pattern, there are large differences in amplitudes (Fig. 3c). Compared to the Fix2023 scenario, the warming by the end of the 21st century in the SSP2-com scenario is decreased over all land regions and most ocean regions, except for the North Atlantic. The largest decrease of >1.2°C is found in the Arctic, followed by the Antarctic, central Asia, western Australia and southern Africa. However, the latest NDCs bring weak temperature mitigation effects of <0.5°C in major emission regions, such as eastern China, India and the USA, which may be attributed to heating effects from co-sourced aerosol emission reductions [25].

Following the increasing global temperature, global mean precipitation continues to increase from 2020 to 2100 under both Fix2023 and SSP2-com scenarios due to enhanced moisture-loading capacity of atmosphere (Fig. 2b). However, global mean precipitation changes exhibit greater variability compared to those of global mean temperature, which may stem from internal climate system sources [6]. Spatially, significant increases in precipitation by the end of the 21st century under both Fix2023 and SSP2-com scenarios mainly occur in equatorial ocean areas, followed by polar zones, relative to the historical period (Fig. 3d and e), which is consistent with previous projections in other scenarios (e.g. SSP1-2.6 and SPP2-4.5) [6]. The strong increase of precipitation between 10°S and 5°N indicates an equatorward shift of the intertropical convergence zone (ITCZ) by the end of the 21st century under both Fix2023 and SSP2-com scenarios, relative to the historical period. However, precipitation in South Asia, India, Australia, the Amazon and most of northern Africa is expected to decrease by the end of the 21st century under both Fix2023 and SSP2-com scenarios, relative to the historical period. Meanwhile, compared to the Fix2023 scenario, the SSP2-com scenario brings more global mean precipitation in the mid-21st century. However, global mean precipitation in both SSP2-com and Fix2023 scenarios is close to that in the late 21st century. This may be attributed to aerosol mitigation in the SSP2-com scenario—lower aerosol loadings will increase cloud-droplet size and shorten cloud lifetime, thereby leading to an increase in precipitation [26]. Compared to the Fix2023 scenario, precipitation over 42% of grid cells shows increasing trends by the end of the 21st century in the SSP2-com scenario. Spatially, there are increasing precipitation levels in China, Southeast Asia and Australia, while decreasing precipitation levels in the USA and Brazil by the end of the 21st century under the SSP2-com scenario, relative to the Fix2023 scenario (Fig. 3f).

### Climate extremes

Compared to mean temperature and precipitation, weather and climate extremes receive more attention, with devasting impacts on ecosystems and human society. Here, using three representative climate extreme events, including heat–humidity extreme, precipitation extreme and wildfire extreme (for details see the Methods section), we further project the changes of climate extremes and assess the extreme climate benefits from nationally determined mitigation efforts.

### Heat–humidity extreme

Extreme heat constitutes a significant threat to human health. Globally, in 2000–19, 489 000 heat-related deaths occurred yearly—45% of these fatalities in Asia and 36% in Europe [27]. Driven by global warming, the frequency of heat extreme days is projected to keep rising throughout the 21st century [28]. Here, using the heat index determined by air temperature and relative humidity, we project the changes of heat–humidity extremes by the end of the 21st century under SSP2-com and Fix2023 scenarios.

**Figure 4.**
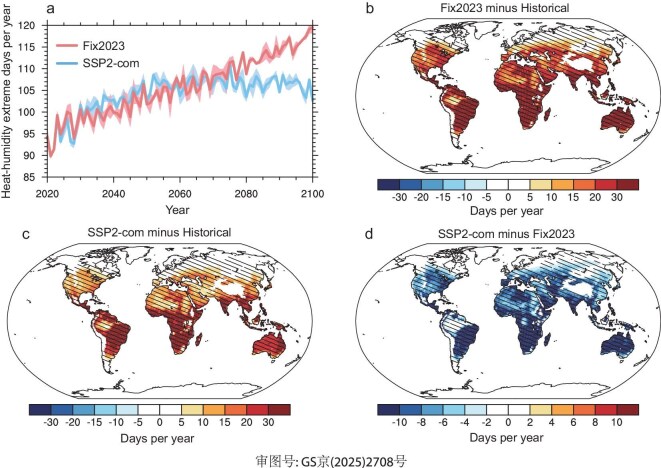
(a) Simulated global mean heat–humidity days during 2020–2100 under Fix2023 and SSP2-com scenarios. The shading represents one standard deviation of three ensemble simulations. (b and c) Simulated annual mean changes of heat–humidity days by the end of the 21st century under Fix2023 (b) and SSP2-com (c) scenarios, relative to the historical period. (d) The differences of simulated annual mean heat–humidity days by the end of the 21st century between SSP2-com and Fix2023 scenarios (SSP2-com minus Fix2023). The hatched regions in panels (b–d) represent that all three ensemble simulations agree on the direction of changes.

In the historical period, the heat–humidity extremes occur with a high frequency of more than 140 days/year in the Amazon, Central and North Africa, India, Southeast Asia and North Australia, followed by approximately 60 days/year in the USA, South Africa, Eastern China and Southern Australia (Fig. S2a). The global mean heat–humidity extreme is projected to increase from 87 days/year in the historical period to 115 days/year by the end of the 21st century under the Fix2023 scenario (Fig. 4a). Regions such as Brazil, Central and South Africa, India, Southeast Asia and Australia are expected to experience the most pronounced increases, exceeding 50 days/year (Fig. 4b). In densely populated regions at mid-latitudes, including the USA, North Africa and Eastern China, the enhancements reach up to 10–20 days/year. Notably, the proportions of heat–humidity extremes in some tropical regions exceed 90% by the end of the 21st century under the Fix2023 scenario (Fig. S2b).

Compared to the Fix2023 scenario, the global mean heat–humidity extreme slightly increases in the near-term future, while it significantly decreases in the long-term future under the SSP2-com scenario (Fig. 4a). The global mean heat–humidity extreme is mitigated by 9 days/year by the end of the 21st century under the SSP2-com scenario. Spatially, the strong mitigating effects of heat–humidity extremes by the end of the 21st century from the SSP2-com scenario are mainly found in the eastern USA, southern South America, Africa, India and Australia (Fig. 4c and d). However, the mitigating effect of heat–humidity extremes by the end of the 21st century from the SSP2-com scenario is limited in China, which will experience the largest emission reductions in the coming decades. This is mainly because the GHG-induced cooling is partly offset by the warming caused by aerosol declines in China.

### Precipitation extreme

**Figure 5.**
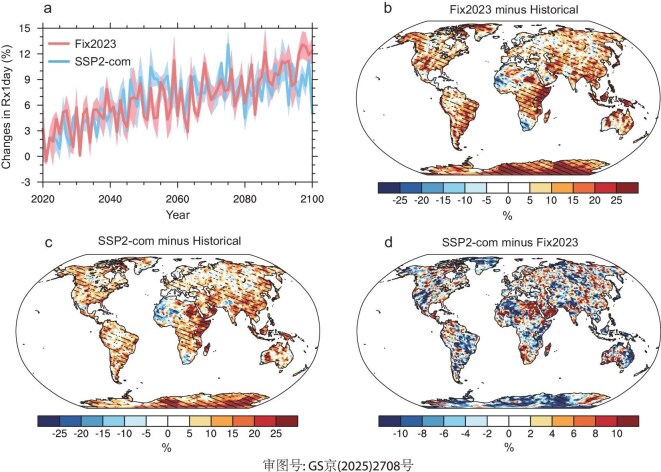
(a) Simulated global mean Rx1day anomalies during 2020–2100 under Fix2023 and SSP2-com scenarios, relative to the historical period. The shading represents one standard deviation of three ensemble simulations. (b and c) Simulated annual mean changes of Rx1day by the end of the 21st century under Fix2023 (b) and SSP2-com (c) scenarios, relative to the historical period. (d) The differences of simulated annual mean Rx1day by the end of the 21st century between SSP2-com and Fix2023 scenarios (SSP2-com minus Fix2023). The hatched regions in panels (b–d) represent that all three ensemble simulations agree on the direction of changes.

Annual maximum daily rainfall (Rx1day), as a critical indicator of extreme precipitation, exhibits high values in monsoon regions, including the eastern USA, South America, western Africa, East Asia and South Asia during 2000–19 (Fig. S3). Under the Fix2023 scenario, global mean Rx1day is projected to increase by 10% by the end of the 21st century, relative to the historical period (Fig. 5a). Spatially, Rx1day shows an increasing trend at 87% of the land grid cells by the end of the 21st century under the Fix2023 scenario (Fig. 5b). The largest increase of Rx1day is found in eastern Africa, where increases may reach up to 20% by the end of the 21st century under the Fix2023 scenario, relative to the historical period. In the USA, India, Australia and eastern China, the Rx1day increases by 5%–10% by the end of the 21st century under the Fix2023 scenario, relative to the historical period. It is noted that although mean precipitation in India, Australia and the Amazon shows decreasing trends, extreme precipitation as indicated by the Rx1day exhibits an increasing trend by the end of the 21st century in the Fix2023 scenario, relative to the historical period.

Compared to the Fix2023 scenario, the global mean Rx1day shows limited changes in the SSP2-com scenario, especially by the end of the 21st century (Fig. 5a). However, there are large regional differences of Rx1day between SSP2-com and Fix2023 scenarios (Fig. 5c and d). Compared to the Fix2023 scenario, the Rx1day over 36% of land grid cells shows an increasing trend, while the Rx1day over 64% of land grid cells shows a decreasing trend by the end of the 21st century in the SSP2-com scenario. The increasing Rx1day mainly occurs in eastern China, India and southern Australia by the end of the 21st century under the SSP2-com scenario, relative to the Fix2023 scenario. It is noted that the Rx1day is mitigated over most land regions in the SSP2-com scenario relative to the Fix2023 scenario, but there are low agreements in the three ensemble simulations, demonstrating large uncertainties in simulating precipitation extreme using ESMs (Fig. 5d).

### Wildfire extreme

**Figure 6.**
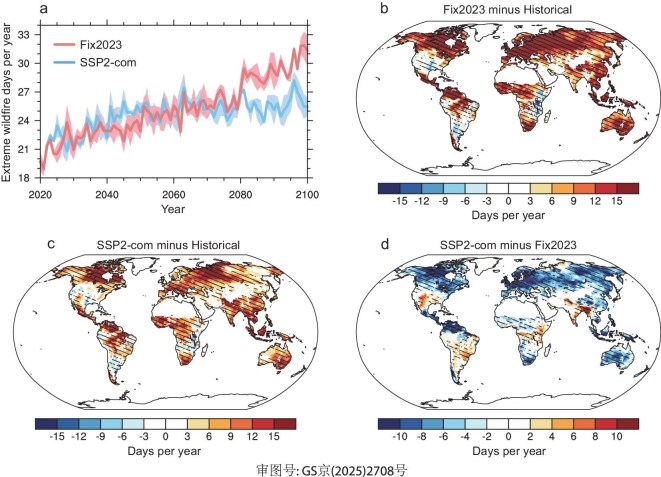
(a) Simulated global mean extreme wildfire days during 2020–2100 under Fix2023 and SSP2-com scenarios. The shading represents one standard deviation of three ensemble simulations. (b and c) Simulated annual mean changes of extreme wildfire days by the end of the 21st century under Fix2023 (b) and SSP2-com (c) scenarios, relative to the historical period. (d) The differences of simulated annual mean extreme wildfire days by the end of the 21st century between SSP2-com and Fix2023 scenarios (SSP2-com minus Fix2023). The hatched regions in panels (b–d) represent that all three ensemble simulations agree on the direction of changes.

In recent years, the world has experienced an intensification of wildfire extreme weather, which has profound negative impacts on the ecosystem, air quality and human health [4]. Here, using the meteorological outputs of the CESM2 to drive the Canadian Forest Fire Danger Rating System (CFFDRS), we project the changes in global wildfire extreme weather under both the SSP2-com and Fix2023 scenarios and assess the avoided wildfire extreme weather resulting from emission reductions outlined in the latest updated NDCs (Fig. 6). Under the Fix2023 scenario, the global mean wildfire extreme weather is projected to continue increasing, reaching 1.6 times by the end of the 21st century, relative to the historical period (Fig. 6a). However, this increase is not uniform across the globe. Spatially, wildfire extreme weather is expected to increase over most land grid cells, particularly in regions that are already major wildfire hotspots, such as Canada, the Amazon, central Africa, south Asia, southern Europe and Australia. Notably, in regions such as Mexico, south Asia and Australia, the increasing wildfire extreme weather exceeds 15 days/year by the end of the 21st century in the Fix2023 scenario (Fig. 6b). However, wildfire extreme weather is expected to slightly decrease in the western USA by the end of the 21st century in the Fix2023 scenario, which may be attributed to increasing precipitation. This highlights the complex interactions between temperature, precipitation and other meteorological variables in shaping future wildfire extreme risks.

Compared to the baseline scenario, wildfire extreme weather shows a slight increase in the near-term future, while it shows a significant decrease in the long-term future at global and regional scales in the SSP2-com scenario (Fig. 6a). On average, global wildfire extreme weather by the end of the 21st century is mitigated from 29.5 days/year in the Fix2023 scenario to 25.7 days/year in the SSP2-com scenario, representing a 21% reduction relative to the historical period. Such mitigating effects are found in 76% of land grid cells and particularly evident in regional hotspots. For example, in Canada, Australia and northern Russia, wildfire extreme weather is expected to decrease by more than 5 days/year by the end of the 21st century in the SSP2-com scenario, relative to the Fix2023 scenario (Fig. 6d). However, not all regions benefit equally from emission reductions. In the western USA and Brazil, Bangladesh and North and South India, wildfire extreme weather is projected to worsen by the end of the 21st century in the SSP2-com scenario, likely due to decreasing precipitation, relative to the Fix2023 scenario.

## DISCUSSION

The climate change projection under SSP scenarios in the CMIP6 has played an irreplaceable role in scientific understanding, policymaking and technological innovation, particularly supporting the released IPCC AR6. However, global anthropogenic emissions have undergone unforeseen changes over the past decade, partly driven by China’s clean air actions and the COVID-19 pandemic [15,16]. Meanwhile, global energy structure has undergone profound changes. For example, the global installed wind power capacity increased by 185%, and the installed photovoltaic capacity increased by 753% from 2013 to 2023. The International Energy Agency (IEA) predicts that the proportion of wind and solar power generation will exceed 60% by 2050 [29]. Therefore, the climate change projection under an updated scenario accounting for emission changes over the past decade and the latest updated NDCs is urgent for the scientific community. Although several studies have explored the climate outcomes of the latest NDCs, these studies are based on simplified climate emulators, which restrict their analytical scope to global average temperature alone [30–32]. In this study, we first present a more plausible global climate change projection based on a complicated ESM, accounting for the spatial pattern of mean climate states and climate extremes during 2020–2100 under a newly developed global emission scenario aligned with China’s carbon neutrality. The projection results demonstrate an encouraging climate mitigation outcome that global warming is more likely to stabilize at 2.0°C through the latest updated NDCs, even in the absence of early extensive deployment of carbon removal technologies.

Based on the daily meteorological outputs, we investigate the future changes of climate extremes with widespread impacts, including heat–humidity extreme, precipitation extreme and wildfire extreme under Fix2023 and SSP2-com scenarios. These findings underscore the importance of the latest updated NDCs in mitigating future global climate extremes, but they also highlight significant regional disparities. These differences are likely driven by the varying responses of meteorological variables, such as temperature and precipitation to emission reductions. While global efforts to reduce GHG emissions are crucial for curbing the overall increase in climate extremes, regional adaptation strategies will be essential to address the specific challenges faced by areas where climate extreme risks such as precipitation and wildfire extremes are expected to rise.

Our study reveals a lagged temperature mitigation benefit lasting for half a century from the latest updated NDCs by comparing the projection results under SSP2-com and Fix2023 scenarios. This can be attributed to strong regional heating effects caused by the sharp reductions in aerosol emission that are co-sourced to CO_2_. In the short term, this may be a pessimistic outcome that an emission mitigation strategy further exacerbates global warming and brings more regional climate extremes. However, climate action centered on emission reductions is undoubtedly the right choice for achieving a long-term sustainable development. If anthropogenic emissions continue at the level of 2023, global warming will reach 2.91°C by 2100. Such a warming climate may trigger multiple climate tipping points [7], leading to potentially irreversible change to human society. In contrast, global warming could be mitigated to 2.05°C and climate extremes will correspondingly decrease by the end of the 21st century through the latest updated NDCs, demonstrating a sustainable climate benefit from rapid climate actions. Moreover, aerosol reductions will contribute to a cleaner near- and long-term future, benefiting both human health and terrestrial ecosystems.

For the newly developed SSP2-com scenario, the CESM2 model projects a temperature rise of 2.05°C for the period 2081–2100, which is lower than the temperature rises of 2.26°C and 3.25°C under SSP1-2.6 and SSP2-4.5 scenarios, respectively (Fig. S4a). Although cumulative CO_2_ emissions under SSP2-com are slightly higher than those under SSP1-2.6, higher scattering aerosol emissions may contribute to a lower warming level. Similarly, the CESM2 model projects a slightly lower global mean precipitation under the SSP2-com scenario compared to the SSP1-2.6 scenario (Fig. S4b). Moreover, the global patterns of changes in relative humidity and sea-level pressure under the SSP2-com scenario resemble those under the SSP1-2.6 and SSP2-4.5 scenarios, but the amplitudes are smaller (Figs S5 and S6).

Several uncertainties are acknowledged here. Firstly, this study uses a single ESM to perform climate projection. While CESM2 is a widely used, advanced ESM in climate change research, there are still simulated biases for mean and extreme climates on regional scales. Additionally, ESMs exhibit differing climate response to emission changes. Moreover, we use only three ensembles to minimize climate internal variability. Therefore, the climate projection from multiple ESMs and large ensembles under the SSP2-com scenario is worthy of further investigation. Secondly, an emission scenario is used to explore possible futures and the effectiveness of climate actions. However, emission scenarios exhibit diversity and uncertainty, given various assumptions regarding socioeconomic development, technological progress and policy interventions. Given the complexity and high computational cost of ESMs, it is not realistic to conduct climate projections for all potential emission pathways. For example, there are 3131 scenarios in the AR6 scenario database. After a screening process, only four common scenarios (SSP5-8.5, SSP3-7.0, SSP2-4.5 and SSP1-2.6) are provided to ESMs for future climate change projection. Similarly, the newly developed SSP2-com scenario aligns with the most likely net-zero pathway for China. Looking ahead, in the future we will design additional scenarios—including those accounting for policy regression and other relevant factors—to support further climate change projections.

Despite these limitations, our study first presents a new climate change projection during 2020–2100 under a more plausible 2.0°C scenario based on CESM2. Our ensemble simulations output the most commonly used daily meteorological variables, including near-surface air temperature (tas), daily maximum near-surface air temperature (tasmax), precipitation (pr), near-surface wind speed (sfcWind) and surface daily minimum relative humidity (hursmin), which will provide an important data foundation for future climate research and governance. In the future, we will extend our study through the following directions. Firstly, this study only focuses on the single climate extreme events. The changes of compound climate extremes such as sequential heatwave and flood and co-occurred drought and heatwave deserve investigation in future work. Moreover, the tipping points receiving widespread concern in climate change discourse are worth evaluating under a more plausible 2.0°C scenario. Secondly, the climate projection plays a pivotal role in advancing impacts, adaptation and vulnerability assessments. However, these assessments require high resolution for climate inputs, such as spatial resolutions of <10 km and temporal frequency of ∼1 h. In the future, using the advanced dynamic and statistical downscaling methods, we will generate high-resolution regional climate projections and share them with the scientific community for climate impact assessments.

## METHODS

### CESM2 model

CESM2 is the newest version of the Coupled Earth System Model developed by the National Center for Atmospheric Research (NCAR), designed to simulate past, present and future climates [33]. CESM2 includes six components: atmosphere, ocean, land, sea/land-ice, river and wave. The atmosphere component uses the Community Atmosphere Model version 6 (CAM6), featuring a horizontal resolution of 1.25° in longitude and 0.9° in latitude and 32 vertical levels. The ocean component utilizes the Parallel Ocean Program version 2 (POP2), which can simulate various processes in the ocean, including the changes in ocean temperature and salinity, ocean circulation, ocean biogeochemistry and exchanges between ocean and atmosphere. The land component uses the Community Land Model version 5 (CLM5), which is capable of simulating the land carbon cycle, hydrological cycle, biogeochemical processes and vegetation–climate feedback. The sea-ice and land-ice components utilize The Los Alamos sea ice model version 5 (CICE5) and the Community Ice Sheet Model version 2 (CISM2), respectively. In the past, CESM2 participated in core simulations and several model comparison projects of CMIP6, helping policymakers and scientists better understand and address climate change. Meanwhile, due to its excellent performance of reproducing historical climate, the CESM2 is widely used for climate projection and climate attribution research [34,35].

### Updated historical emissions

The historical simulations in CMIP6 are based on global anthropogenic emissions from the Community Emissions Data System (CEDS v2016-07-26) inventory [36]. However, due to the long development times of global bottom-up inventories, the global CEDS v2016-07-26 fails to capture emission trends over recent years in some regions undergoing rapid change such as China, North America, Europe, India and Africa. For example, anthropogenic emissions in China show large declines during 2006–15, but these changes in CEDS v2016-07-26 are comparatively small, or even show opposite trends [37], leading to inaccurate regional climate simulations in CMIP6 models [38]. Recently, the CEDS inventory is updated to v2021-04-21 with improvements in regional inventory datasets and extending to 2019. Compared to CEDS v2016-07-26, the CEDS v2021-04-21 shows lower global anthropogenic emissions, including CO_2_, SO_2_, black carbon (BC) and organic carbon (OC) (black vs. grey lines in Fig. S7), with the reduction being particularly notable in East Asia (Fig. S8). Especially for global BC and OC emissions, the positive trends after 2010 in CEDS v2016-07-26 have been updated to negative trends in CEDS v2021-04-21. Considering the large improvements in global anthropogenic emissions, we replace the CEDS v2016-07-26 inventory with the CEDS v2021-04-21 inventory in CESM2 to acquire accurate climate simulations in the historical period.

### Future climate scenarios

Climate scenarios are essential for future climate projections. The widely used SSP scenarios in CMIP6, supporting the IPCC AR6, are now outdated by almost a decade. Considering unforeseen changes in anthropogenic emissions in the past decade and the latest updated NDCs from major emitting countries towards carbon neutrality, the climate projection under current SSP scenarios faces limitations to support the ongoing IPCC AR7. Recently, Zhong *et al.* developed a new global scenario (named SSP2-com), accounting for up-to-date anthropogenic emissions and the latest updated NDCs around the world, including China’s dual carbon pledge [20]. The SSP2-com scenario is built upon global updated NDCs of all countries and global net-zero pledges. It assumes that these NDC commitments will be maintained until 2030, with emissions trajectories aligning with unconditional NDC targets. Beyond 2030, efforts are set to be scaled up to achieve global net-zero CO_2_ emissions around 2070, supported by accelerated decarbonization. In the SSP2-com scenario, global CO_2_ emission achieves net zero by 2072 (Fig. S7a), which is aligned with the CMIP7 ScenarioMIP low emission scenario net-zero target. The Asia–Pacific region, currently the largest contributor to global CO_2_ emissions, is projected to be the primary driver of emissions reductions by 2100—with its reductions set to account for 42% of total global reductions [20]. For China, CO_2_ emissions peak at about 12.8 GtCO_2_ around 2028–29, with emissions expected to drop to about 11.2 GtCO_2_ by 2035, continue to decrease to 3.6 GtCO_2_ by 2050, and finally reach 0.9 GtCO_2_ by 2060 after peaking. Sectorally, the energy sector will contribute the most to these reductions (66%), followed by industry (21%) and transport (9%). The developed countries region, the second-largest contributor to global CO_2_ emissions, is projected to account for 29% of total global reductions, with primary drivers being the energy and transport sectors. As for other greenhouse gases (e.g. CH_4_, N_2_O) and air pollutants, their future emissions trajectories are derived using the quantile rolling window (QRW) technique, which has been applied in the AR6 WGIII report [39]. The QRW technique derives a relationship between emissions species time series in the ‘infiller’ database—by segmenting these into rolling windows and calculating quantiles—then applies this relationship to a ‘target’ database. In the SSP2-com scenario, global SO_2_, BC and OC emissions are expected to decline by 75%, 74% and 83% by 2100, relative to 2020, respectively (Fig. S7b–d). Regionally, these air pollutants exhibit a large decline by 2100 in East Asia, followed by a relatively smaller decline in Europe and the USA. Compared to scenarios from the ScenarioMIP-CMIP6, the emission levels of the SSP2-com scenario fall between SSP1-2.6 and SSP2-4.5 scenarios, with a closer alignment to the SSP1-2.6 scenario (Fig. S7). Using three simplified climate emulators, Zhong *et al.* reported a GMST rise of 2.04°C during 2091–2100 under the SSP2-com scenario, relevant to the Paris Agreement target [20]. To further assess the potential climate benefits from such a more plausible 2°C global scenario, we set a baseline scenario (named Fix2023) where future global anthropogenic emissions including those of aerosols and GHGs during 2023–2100 are fixed in 2023, close to the medium scenario in the ScenarioMIP-CMIP7 proposal [18]. In the ScenarioMIP-CMIP7 proposal, the medium emission scenario serves as a benchmark representing the consequences of continuing current climate policies. While this scenario is unrealistic, it is intended to explore the relative benefits of taking further climate actions.

### Model experiments

To acquire more plausible climate change in the 21st century and quantify the potential climate benefits from the latest updated NDCs, we use the fully coupled CESM2.1.3 to conduct three sets of transient climate simulations: (i) updated historical simulation; this simulation is restarted from 1990 and ended in 2019 based on the global CEDS v2021-04-21 inventory; (ii) Fix2023 simulation; this is a baseline climate projection from 2020 to 2100 based on global anthropogenic emissions from the Fix2023 scenario; and (iii) SSP2-com simulation; this is a more plausible climate projection from 2020 to 2100 based on global anthropogenic emissions from the SSP2-com scenario. Each set includes three ensemble simulations, which use the same pathway of GHG and aerosol forcing but with different atmospheric initial conditions. All simulations are driven by CO_2_ emissions rather than CO_2_ concentration. The temporal–spatial CO_2_ concentration is calculated based on the carbon cycle module of CESM2.1.3, which further affects climate change. Moreover, CH_4_ and N_2_O concentrations are simulated based on the MAGICC simplified climate emulator (Fig. S9) and further used as external forcing to drive CESM2.1.3. The widely used five daily variables, including tas, tasmax, pr, sfcWind and hursmin, are output from each simulation, which allows acquisition of a more plausible climate change projection and assessment of the potential climate benefits from the latest updated NDCs.

### Estimate of warming level

To minimize the bias of ESMs in projecting the warming level by the end of the 21st century, we assume that the simulated global warming during 2011–20 above pre-industrial levels aligns with the observation of 1.09°C reported by IPCC AR6. Essentially, this is equivalent to discussing the remaining space of future GMST rise on the basis of the current GMST rise of 1.09°C. Therefore, the projected warming level in this study is calculated by adding simulated GMST changes ($\Delta $GMST) during 2020–2100 relative to the decade average during 2011–20 on the observed GMST rise of 1.09°C during 2011–20.

### Definitions of climate extremes

To investigate the future changes in climate and weather extremes, three types of climate extreme events are defined here: heat–humidity extreme, precipitation extreme and wildfire extreme. The heat–humidity extreme event is defined by a daily maximum heat index exceeding 32°C in this study. The heat index determined by air temperature and relative humidity indicates the ‘feel-like’ temperature of the human body and serves as a common index for assessing heat-related health risks [40]. Here, we calculate the daily maximum heat index based on the meteorological variables tasmax and hursmin, following the formula by the National Weather Service of the USA (https://www.wpc.ncep.noaa.gov/html/heatindex_equation.shtml). Precipitation extreme is represented by annual maximum Rx1day. Wildfire extreme is defined as days exceeding the 95th percentile of the Fire Weather Index (FWI) in the historical period (Fig. S10). The FWI is derived from the CFFDRS, which is a widely used numerical indicator for potential wildfire intensity. The standard calculation of FWI requires 24 h accumulated pr and tas, relative humidity and sfcWind at noon local time. Given the difficulty of outputting hourly variables from ESMs, we use tasmax, pr, hursmin and sfcWind to calculate FWI following previous studies [41,42]. Moreover, we apply a mask to remove non-vegetated grid cells with the land cover dataset of the moderate-resolution imaging spectroradiometer (MODIS).

### Validation of CESM2.1.3

We evaluate the present-day mean climate and extreme climate from updated CESM2 historical simulations against the fifth-generation European Centre for Medium-Range Weather Forecasts (ECMWF) reanalysis (ERA5) (Figs S11 and S12). The results show that the CESM2 model can reasonably reproduce the global spatial patterns of near-surface temperature and precipitation in the historical period (2000–19), with high spatial coefficients of 0.99 and 0.93 and low normalized mean biases of 5.7% and 0.5%, respectively (Fig. S11). Spatially, the CESM2 model exhibits relatively limited biases in near-surface temperature across all grids, except for the central USA and northern India. For precipitation, small simulated biases occur in mid-to-high latitudes, while relatively large simulated biases are concentrated in tropical regions. Similar to the mean climate, the CESM2 model also reasonably captures the global spatial patterns of extreme climate, but with certain biases on regional scales (Fig. S12). For heat–humidity extreme, the CESM2 model overestimates the frequencies in the eastern USA, northern South America and eastern China, while underestimating it in Brazil and northern Africa. For Rx1day, the CESM2 model shows slight overestimation in the southeastern Qinghai Tibet Plateau and northeastern Asia. For the 95th percentile of FWI, the CESM2 model exhibits overestimations in the central USA, South America and northern India.

## Supplementary Material

nwaf545_Supplemental_File

## Data Availability

The ERA5 reanalysis used for model evaluation is publicly available at https://cds.climate.copernicus.eu/datasets. The SSP2-com forcing is publicly available at http://meicmodel.org.cn/?page_id=2903. The CEDS v2021-04-21 is publicly available at https://data.pnnl.gov/dataset/CEDS-4-21-21. The CESM2 outputs under SSP2-com and Fix2023 scenarios are available upon request from the corresponding author Z.W.
