# Supplementary material for: Climate mitigation outcomes from China-led emission reductions toward global carbon neutrality
Source: Natl Sci Rev. 2025 Dec 9;13(1):nwaf545. doi: 10.1093/nsr/nwaf545 (PMC12796822; doi:10.1093/nsr/nwaf545)
Supplement: nwaf545_Supplemental_File [file nwaf545_supplemental_file.pdf]

Supporting information for

Climate Mitigation Outcomes from China-led Emission Reductions toward Global Carbon Neutrality

Yadong Lei[1], Zhili Wang[1]*, Junting Zhong[1], Xiaochao Yu[1], Lifeng Guo[1], Chenguang Tian[2], Lei Li[1], Yixiong Lu[3], Da Zhang[4], Lin Liu[1], Deying Wang[1], Huizheng Che[1], Xiaoye Zhang[1]*

[1]State Key Laboratory of Severe Weather & Key Laboratory of Atmospheric Chemistry of CMA, Chinese Academy of Meteorological Sciences, Beijing, 100081, China

[2]School of Environmental Science and Engineering, Nanjing University of Information Science & Technology, Nanjing, 210044, China

[3]CMA Earth System Modeling and Prediction Centre, China Meteorological Administration, Beijing, 100081, China

[4]Tsinghua-CTG Joint Center for Climate Governance and Low-carbon Transformation, Tsinghua University, Beijing 100086, China

Correspondence to: Zhili Wang (wangzl@cma.gov.cn) and Xiaoye Zhang (xiaoye@cma.gov.cn)

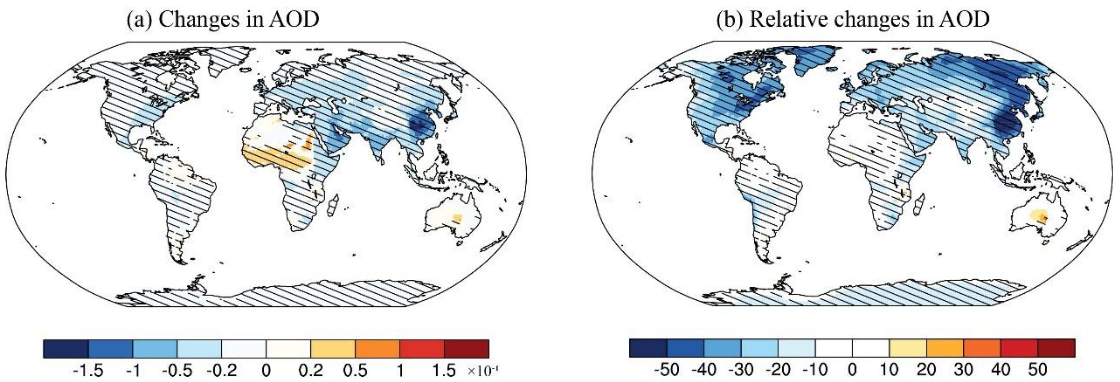

Figure S1 Changes in aerosol optical depth (AOD). The absolute **(a)** and relative **(b**, Unit: %**)** changes in AOD by the end of 21st century (2081-2100) under the SSP2-com scenario, relative to the historical period. The hatched regions represent that all three ensemble simulations agree on the direction of changes.

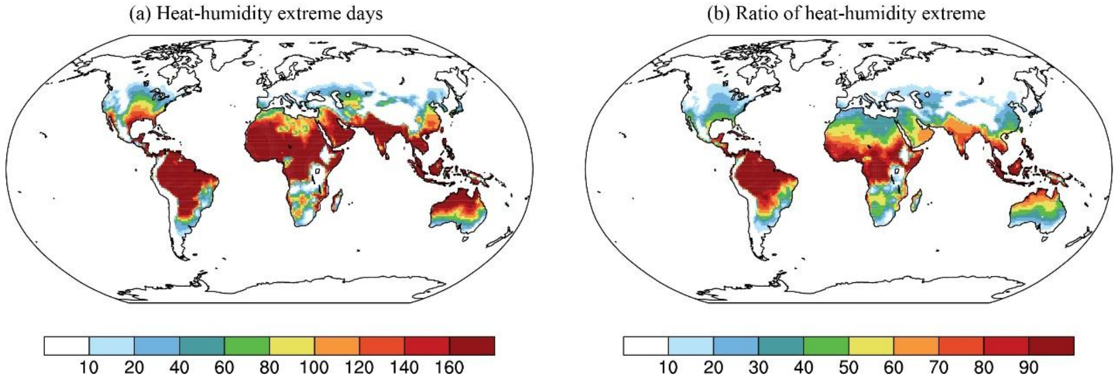

Figure S2 Changes in heat-humidity extreme days. **(a)** Annual mean heat-humidity extreme days (Unit: days yr^{-1}) in the historical period. **(b)** The annual mean ratio (Unit: %) of heat-humidity extreme days by the end of 21st century under the Fix2023 scenario.

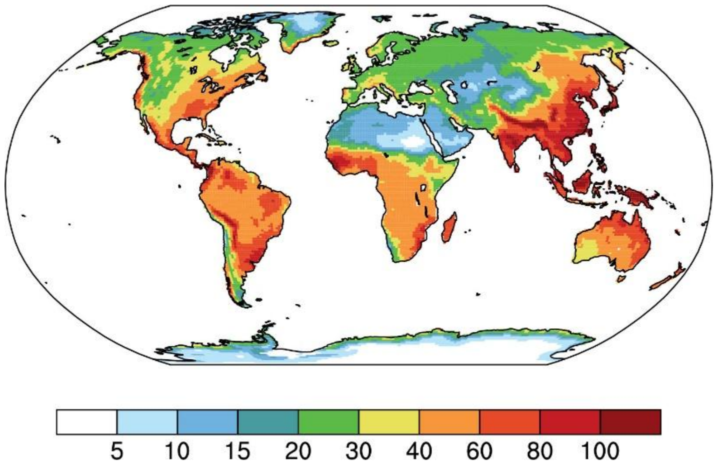

Figure S3 The annual maximum daily rainfall (Rx1day, Unit: mm day^{-1}) in the historical period.

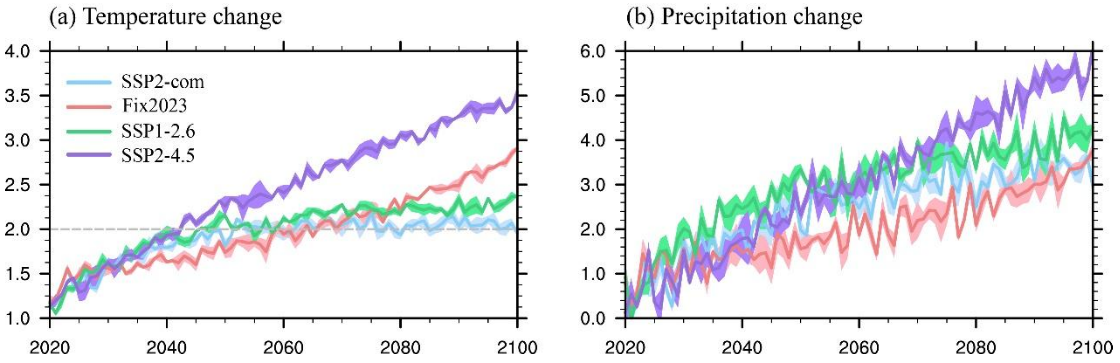

Figure S4 Comparisons of temperature and precipitation changes among different emission scenarios. **(a)** Simulated global mean surface air temperature anomalies (Units: °C) relative to 1850-1900 during 2020-2100 under Fix2023, SSP2-com, SSP1-2.6 and SSP2-4.5 scenarios. **(b)** Simulated global mean precipitation anomalies (Units: %) during 2020-2100 under Fix2023, SSP2-com, SSP1-2.6 and SSP2-4.5 scenarios, relative to the historical period (2000-2019). Notably, different ensemble sets are used for comparison. The CESM2 model in CMIP6 only offers three ensemble members—r4i1p1f1, r10i1p1f1, and r11i1p1f1—which differ from ours (r1i1p1f1, r2i1p1f1, and r3i1p1f1).

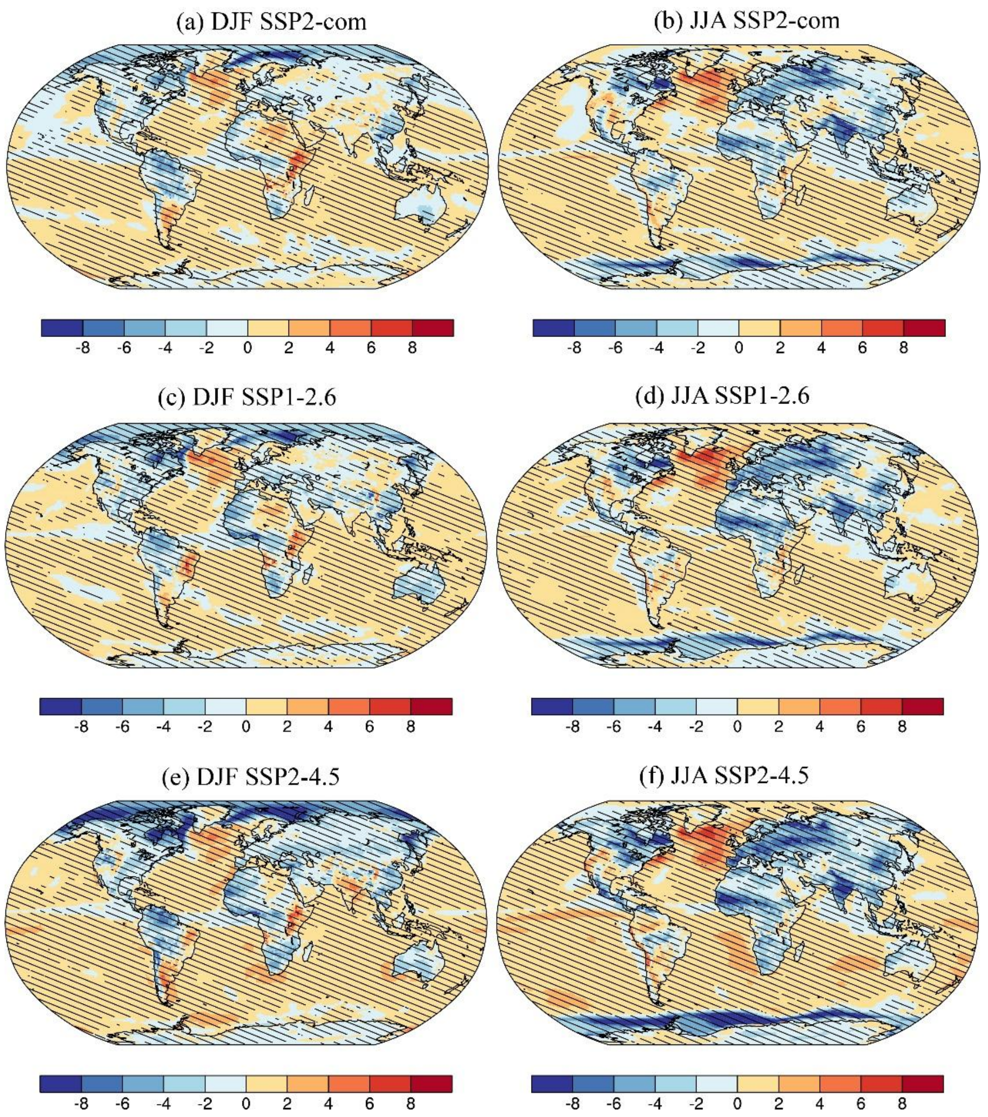

Figure S5 Comparisons of relative humidity changes (Units: %) among different emission scenarios. **(a, c, e)** Simulated changes of December-January-February (DJF) relative humidity during 2081-2100 under SSP2-com, SSP1-2.6 and SSP2-4.5 scenarios, relative to the historical period (2000-2019). **(b, d, f)** Simulated changes of June-July-August (JJA) relative humidity during 2081-2100 under SSP2-com, SSP1-2.6 and SSP2-4.5 scenarios, relative to the historical period (2000-2019). Notably, different ensemble sets are used for comparison. The CESM2 model in CMIP6 only offers three ensemble members—r4i1p1f1, r10i1p1f1, and r11i1p1f1—which differ from ours (r1i1p1f1, r2i1p1f1, and r3i1p1f1).

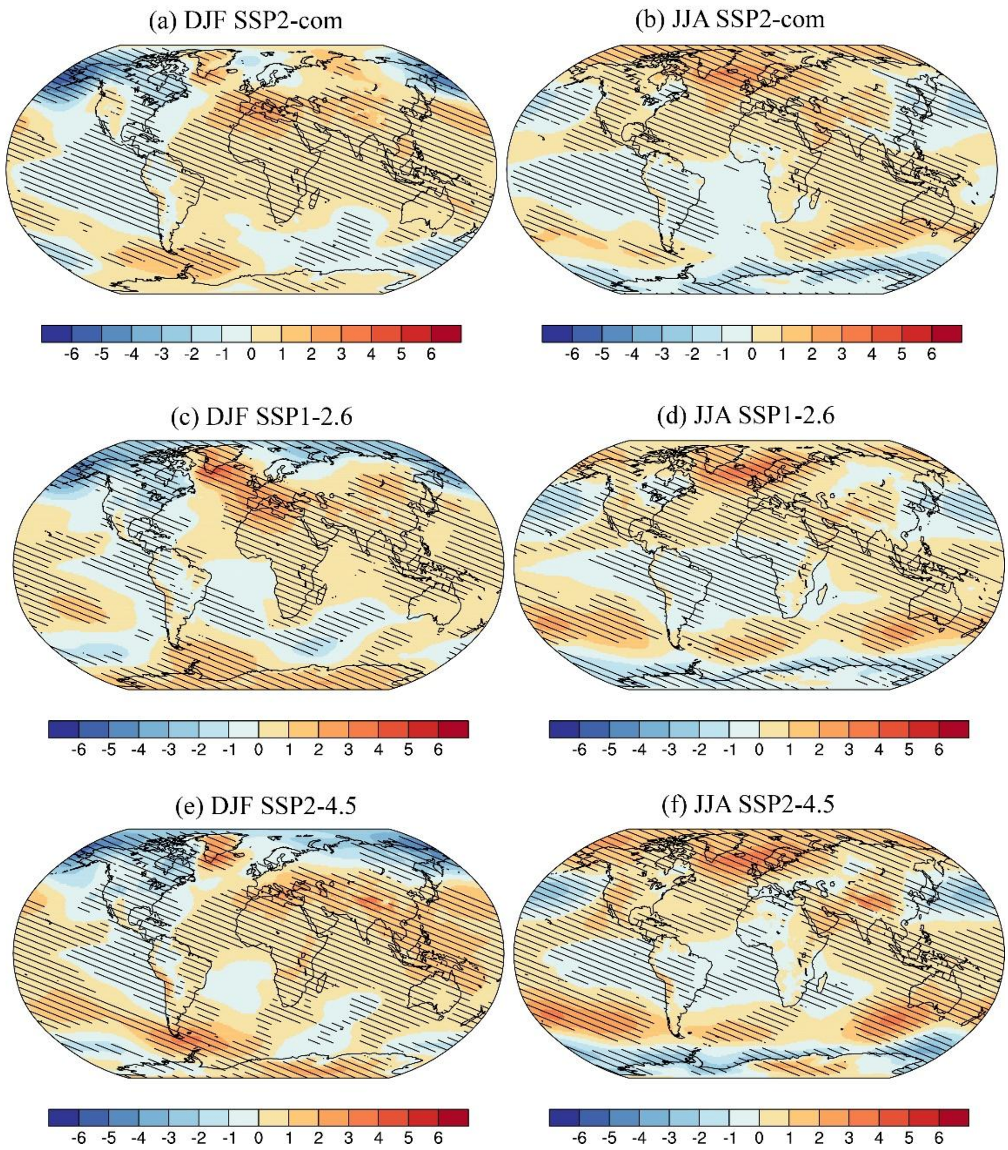

Figure S6 Comparisons of sea level pressure changes (Units: hPa) among different emission scenarios. **(a, c, e)** Simulated changes of December-January-February (DJF) sea level pressure during 2081-2100 under SSP2-com, SSP1-2.6 and SSP2-4.5 scenarios, relative to the historical period (2000-2019). **(b, d, f)** Simulated changes of June-July-August (JJA) sea level pressure during 2081-2100 under SSP2-com, SSP1-2.6 and SSP2-4.5 scenarios, relative to the historical period (2000-2019). Notably, different ensemble sets are used for comparison. The CESM2 model in CMIP6 only offers three ensemble members—r4i1p1f1, r10i1p1f1, and r11i1p1f1—which differ from ours (r1i1p1f1, r2i1p1f1, and r3i1p1f1).

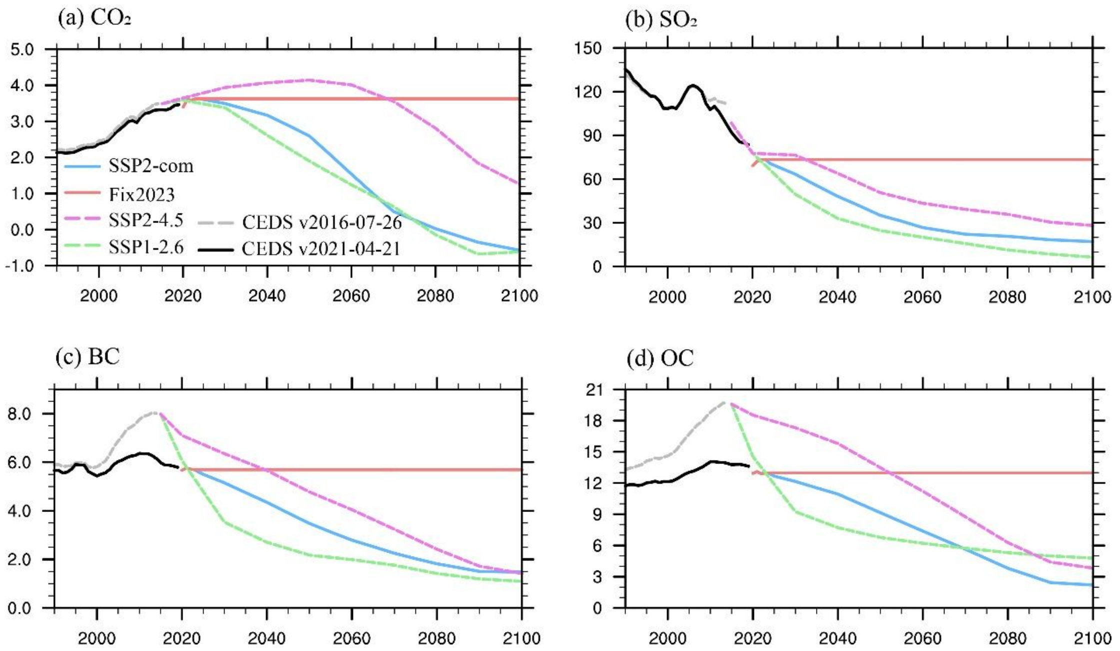

Figure S7 The historical and future changes in global anthropogenic emissions. **(a-d)** Global emission changes in carbon dioxide (CO_2, Units: $\times10^4$ Mt), sulfur dioxide (SO_2, Units: Mt) black carbon (BC, Units: Mt) and organic carbon (OC, Units: Mt) during 1990-2100.

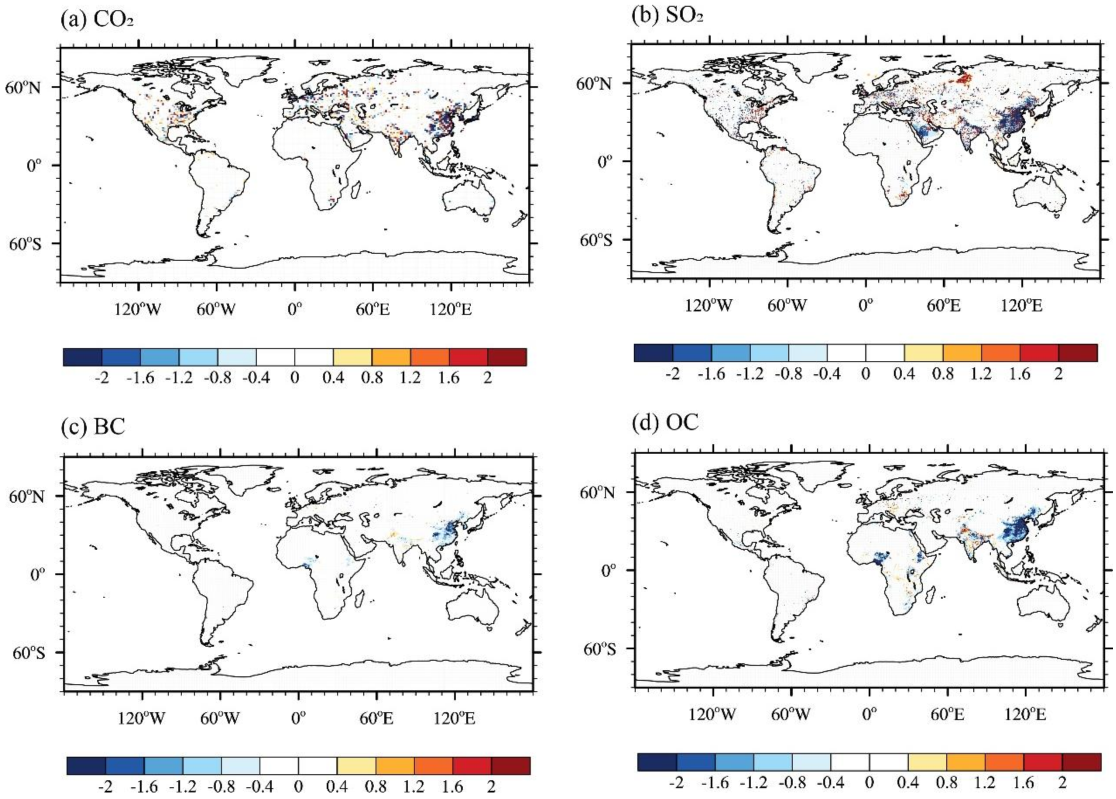

Figure S8 The emissions differences of CO_2 (Units: 10^{-8} kg m^{-2} s^{-1}; **a**), SO_2 (Units: 10^{-11} kg m^{-2} s^{-1}; **b**), BC (Units: 10^{-11} kg m^{-2} s^{-1}; **c**) and OC (Units: 10^{-11} kg m^{-2} s^{-1}; **d**) in 2014 between CEDS v2016-07-26 and CEDS v2021-04-21 (CEDS v2021-04-21 minus CEDS v2016-07-26).

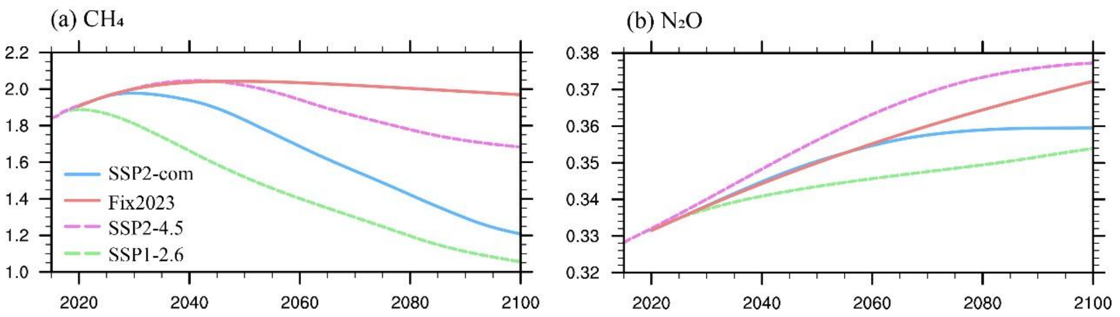

Figure S9 The changes in methane (CH_4, Units: ppmv) and nitrous oxide (N_2O, Units: ppmv) concentrations during 2020-2100 under SSP2-com, Fix2023, SSP1-2.6 and SSP2-4.5 scenarios.

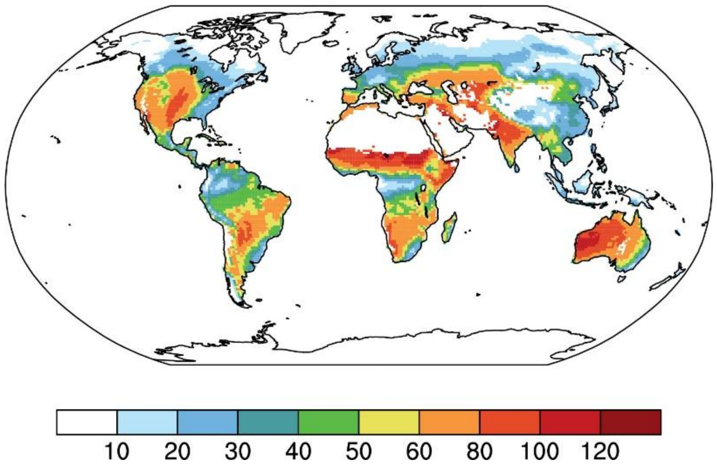

Figure S10 The 95th percentile value of Fire Weather Index (FWI, Unitless) in the historical period (2000-2019).

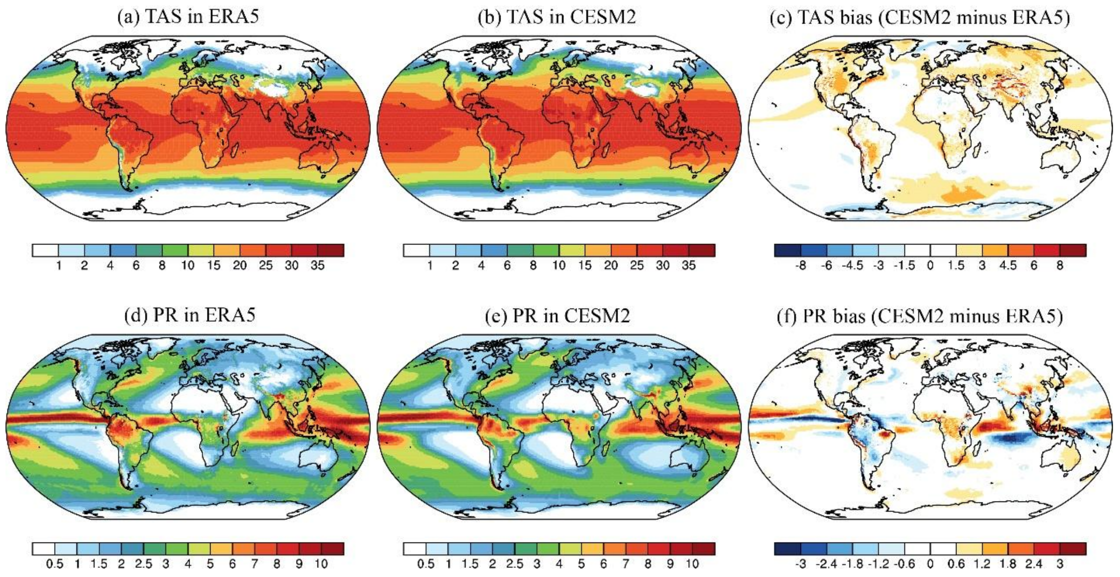

Figure S11 The observed **(a, d)** and simulated **(b, e)** climatology of near-surface temperature **(a, b**; Unit: °C**)** and precipitation **(d, e**; Unit: mm day^{-1}**)** in the historical period (2000-2019) using CESM2 with updated anthropogenic emission inventory. **(c, f)** The difference of near-surface temperature and precipitation between simulation and observation (CESM2 minus ERA5).

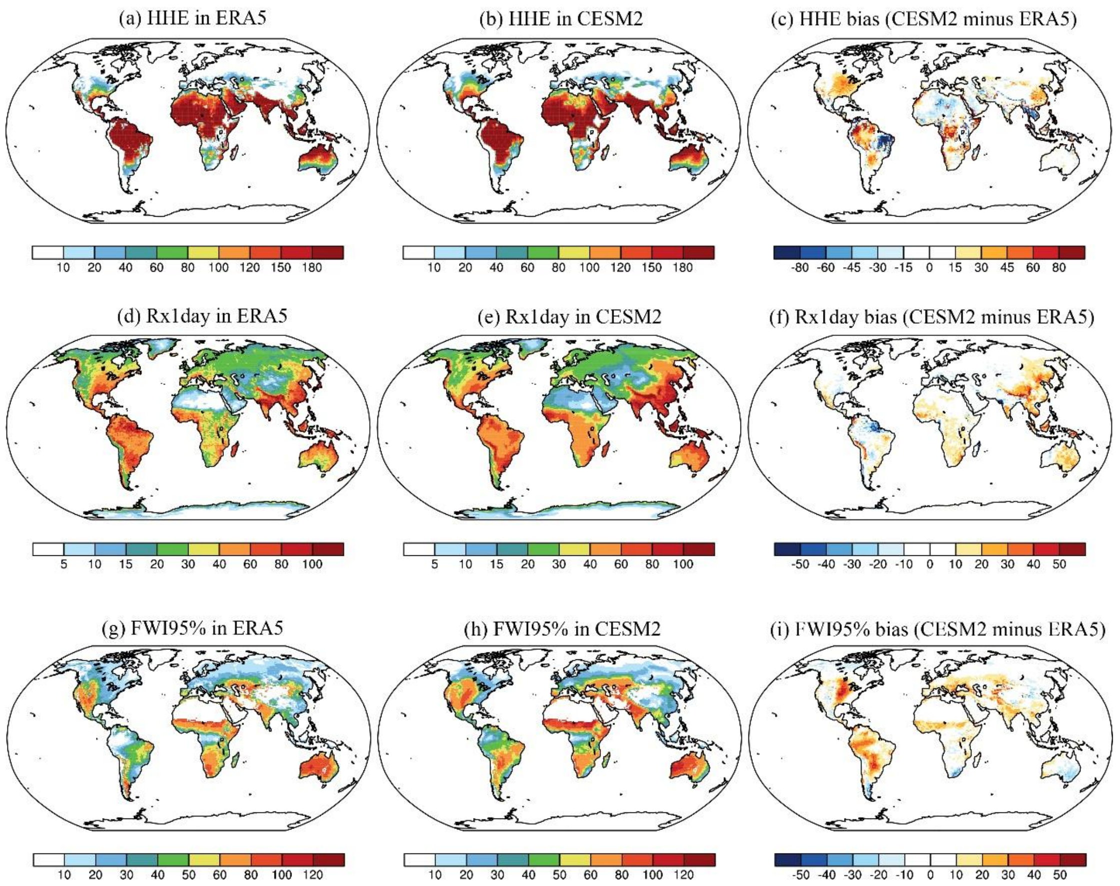

Figure S12 The observed **(a, d, g)** and simulated **(b, e, h)** heat-humidity extreme frequency **(a, b, c**; Unit: days yr^{-1}**)**, Rx1day **(d, e, f**; Unit: mm day^{-1}**)** and 95th percentile value of FWI **(g, h, i**; Unitless**)** in the historical period (2000-2019) using CESM2 with updated anthropogenic emission inventory. **(c, f, i)** The difference of heat-humidity extreme frequency, Rx1day and 95th percentile value of FWI between simulation and observation (CESM2 minus ERA5).
